# Transitional Vertebra and Spina Bifida Occulta Related with Chronic Low Back Pain in a Young Patient

**DOI:** 10.7759/cureus.837

**Published:** 2016-10-19

**Authors:** Maryam Kundi, Maham Habib, Sumbal Babar, Asif K Kundi, Salman Assad, Amjad Sheikh

**Affiliations:** 1 Department of Internal Medicine, Carthage Area Hospital, New York, USA; 2 Department of Medicine, Shifa Tameer-e-Millat University, Islamabad, Pakistan

**Keywords:** bertolotti’s syndrome, lumbosacral transitional vertebrae, low back pain, pseudo-articulation

## Abstract

Bertolotti’s syndrome (BS) must be considered as a differential diagnosis in a young patient presenting with low back pain (LBP). We present a case of a 26-year-old male complaining of mild chronic LBP for six years, radiating to his left thigh for the past six months. He has been taking non-steroidal anti-inflammatory drugs (NSAIDs) with skeletal muscle relaxants for pain relief. The X-ray and computed tomography (CT) imagings showed congenital enlargement of the left transverse process of the fifth lumbar (L5) vertebra forming pseudo-articulation with the sacrum and unilateral pars interarticularis defect at the L4 level on the left side, respectively. He has managed with gabapentin 100 mg three times a day for his neuropathic left leg pain. On follow-up, the patient reported that his pain has improved with gabapentin and it decreased from 8/10 to 4/10 on the visual analogue scale.

## Introduction

Bertolotti's syndrome (BS) is described by the presence of a variation of the fifth lumbar (L5) vertebra with a large transverse process which is combined or articulated with either iliac crest or sacral basis leading to chronic persistent back pain [1]. Lumbar sacral transitional vertebrae (LSTV) are congenital spinal anomalies defined as either sacralisation of the lowest lumbar segment or lumbarisation of a most superior sacral segment of the spine. Bertolotti, an Italian surgeon stated in 1917 that these abnormal vertebrae may produce low back pain (LBP) due to arthritic changes occurring at the site of pseudoarthrosis. The syndrome is said to affect four to eight percent of the population. LSTV is reported to be more prevalent in men than in women [2]. We report a case of a young patient with chronic low back pain radiating to his left thigh along the distribution of L4 dermatome. Informed consent was obtained from the patient for this study.

## Case presentation

A 26-year-old male presented with a five-year history of mild chronic LBP. The patient had a history of ulcerative colitis, diverticulitis, hypertriglyceridemia, depressive mood disorder, and hypogonadism with erectile dysfunction. His diverticulosis was well controlled with a high fiber diet and hyoscyamine when needed. Initially, the back pain did not radiate to the legs. He had been taking naproxen (non-steroidal anti-inflammatory drug) and cyclobenzaprine (skeletal muscle relaxant) since the onset of LBP, which according to the patient, was working well for him. On a recent visit, he reported having numbness, tingling, and worsening aching pain over the left thigh and left knee for the last six months. The numbness occurs after a few minutes of sitting or lying down and sensations return after a few seconds. The patient denied any previous injury to the left leg, restricted range of motions, swelling or tenderness. We prescribed gabapentin 100 mg three times a day for his neuropathic left leg pain. On follow-up, the patient reported that his pain has improved with gabapentin and it has decreased from 8/10 to 4/10 on a visual analogue scale. In the past, he has been hospitalized multiple times for lumbago, colitis, urinary tract infection (UTI), and depressive disorder. He was previously advised physical therapy for LBP but he went against medical advice and opted not to undergo physical therapy. His back pain was initially misdiagnosed as muscle sprain due to heavy weight lifting at the gym.

The physical examination revealed a healthy, white, small-built male with a normal gait. The patient was vitally stable. The spinal contour was normal. No motor or sensory deficits were found in the lumbosacral region. On palpation, there was no spinal or para-spinal muscle tenderness but a dimple was felt over the left side of the L5-S1 region. Costovertebral angle tenderness (CVAT) was negative. On examination of lower extremities, the tone and muscle bulk were normal. There was a loss of sensations over the left anterior thigh along the L4 dermatomal distribution. The power was 5/5 and the deep tendon reflexes (DTRs) were brisk and symmetrical bilaterally. The Lasègue test/straight leg raise test was negative. The laboratory investigations are shown in Table 1.

**Table 1:** The patient’s laboratory findings

Laboratory Variables	Values
Serum calcium	10.2 mg/dl
25-OH Vitamin D	38.6 ng/mL
Thyroid stimulating hormone (TSH)	3.57 mIU/L
Free T4	0.96 ng/dL
Serum sodium	144 mEq/L
Serum potassium	4.2 mEq/L
Blood urea nitrogen (BUN)	9 mg/dL
Serum trigylcerides	175 mg/dL
Serum cholesterol	192 mg/dL

**Figure 1:**
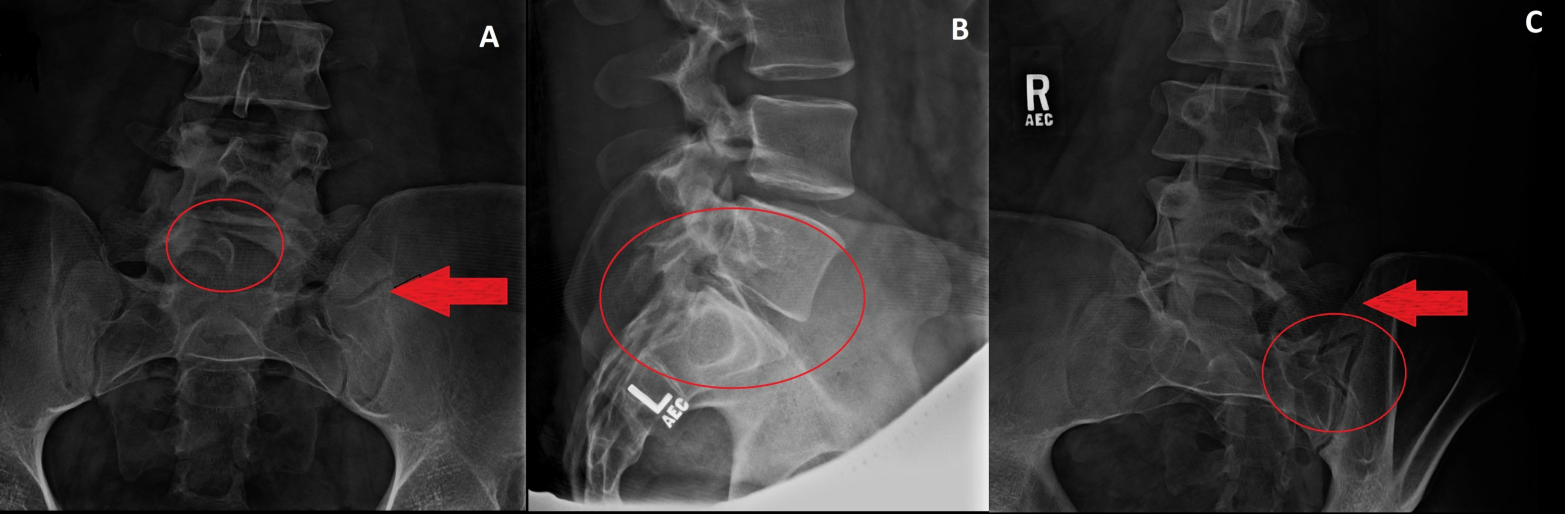
X-ray of LS [A] Anteroposterior (AP) view: The red arrow shows enlargenment of the left transverse process of L5 vertebra forming pseudo-articulation with the sacrum. The red circle shows spina bifida occulta. [B, C] Left lateral and right lateral views: the red arrow [C] shows enlargement of the transverse process of L5. The red circles [B, C] demonstrate pseudo-articulation of the L5 transverse process with the sacrum.

An X-ray lumbosacral (LS) of the spine showed congenital enlargement of the left transverse process of L5 forming pseudoarticulation with the sacrum. Spina bifida occulta of the L5-S1 were incidental findings. The vertebral body heights, interspacing, and alignment were found to be normal. There was no spondylolysis or spondylolisthesis (Figure 1).

**Figure 2:**
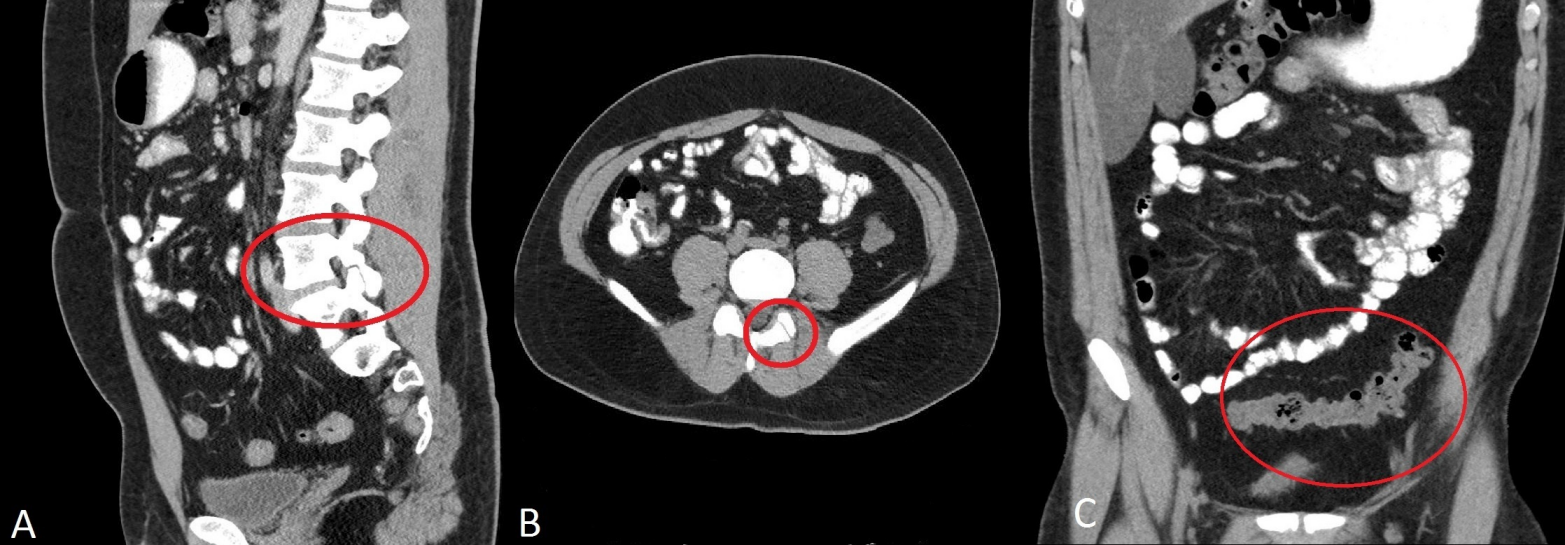
CT scan of abdomen and pelvis [A] Sagittal view [B] Axial view show unilateral pars interarticularis defect at the L4 level on the left side. [C] Coronal view demonstrates mild diverticulosis in the sigmoid colon without any evidence of diverticulitis.

A CT scan of the abdomen and the pelvis was performed for his abdominal pain. We administered 75 ml of Optiray 350 intravenously, and the imaging demonstrated mild diverticulosis in the sigmoid colon without any evidence of diverticulitis. There was a unilateral pars interarticularis defect at the L4 level on the left side (Figure 2).

## Discussion

**Figure 3:**
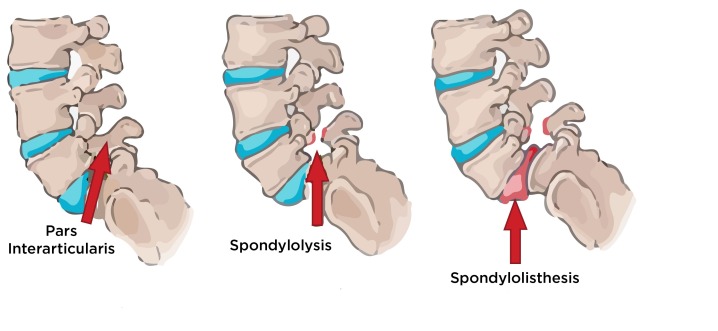
Anatomical location of pars interarticularis and stages leading to spondylolisthesis Spondylolysis is the medical term for a spine fracture or defect that occurs at the region of the pars interarticularis. The pars interarticularis is the region between the facet joints of the spine and more specifically the junction of the superior facet and the lamina. Spondylolysis is thought to be caused by repeated strains that damage the lower spine over time. Repeated strains can eventually lead to an overuse injury in the pars interarticularis. The most common location for this to occur is in the lowest vertebra of the spine. It is common for the defect to occur on both sides. When this happens, the vertebra is no longer held firmly in place by the facet joints on the back of the ring. As a result, the vertebra is free to slip forward over the one below. This slippage which is closely related to spondylolysis is called spondylolisthesis.

Low back pain (LBP) is the fifth most common reason for all physician visits and is the second most common symptomatic reason [3]. LBP has a major economic impact in the United States, with total costs related to this condition exceeding $100 billion per year [3]. In a study done by Quinlan, et al., the overall incidence of BS was found to be 4.6%. It was present in 11.4% of the under-30 age group. Disc generation was seen in 568 of those patients [4]. Kurt EE, et al. [5] found a relationship between the frequency of lumbar disc herniation (LDH) and LSTV in young patients with chronic LBP between the ages of 20 and 40. An increased prevalence of disc herniation above the transitional L5 vertebra has been found in patients with LBP. A decreased prevalence of disc protrusion or extrusion was found in the disc below the transitional vertebra. The authors suggested that this phenomenon was due to hypermobility and abnormal torque of the intervertebral space above the transitional vertebra and that there was less degenerative change at the level below because the anomalous articulations allow less movement between the L5 and S1 vertebrae [6]. As seen in our case, the patient experienced pain and sensory deficit along the distribution of L4 dermatome while the region supplied by L5 was spared. No difference in the prevalence of spondylolysis or spondylolisthesis has been reported among patients with transitional vertebra and group controls [1]. Pars interarticularis defect may lead to spondylolysis and spondylolisthesis (Figure 3).

There are four types of LSTV according to Castellvi's classification (Table 2).

**Table 2:** Castellvi’s classification [7]

Castellvi’s Classification	Description
Type I	Dysplastic transverse process with height > 90 mm
Type II	Incomplete lumbarisation/sacralisation
Type III	Complete lumbarisation/sacralisation with complete fusion with the neighboring sacral basis
Type IV	Mixed

Our case has type II LSTV, as an asymmetric pseudoarthrosis with minor arthritic changes was found on X-Ray. A diarthrosis between transverse process and sacrum (type II transitional vertebra) is related to LBP [2]. Nardo L, et al. revealed that LSTV types II and IV positively correlate with the prevalence and severity of LBP and buttock pain [8]. Whether an abnormal vertebra produces symptoms of LBP is still a controversial subject. Many authors have suggested that lower back pain is related to the lumbar-sacral transitional vertebra. Castellvi, et al. [7] found only 21% prevalence of the transitional vertebra in patients with back pain and sciatica. Clinical features correlated with a plain X-ray of the lumbosacral spine (LS) is usually sufficient for the diagnosis of BS without radicular features. However, the presence of radiculopathy requires a magnetic resonance imaging (MRI) for evaluation of a prolapsed intervertebral disc.

Although several treatment options are available in the literature to address this syndrome, there is no consensus regarding the best treatment modality till date. Topical and oral medications, intrathecal injections and transcutaneous electrical nerve stimulation therapies are commonly used non-operative modalities. In the emergency department, opioids are among the most commonly used agents (61.7%) followed by NSAIDs (49.5%) for LBP. The most commonly used combination is NSAIDs with skeletal muscle relaxants (26.2%), as seen in our case, followed by NSAIDs with opioids (25.9%) [9]. Adequate immediate pain relief has been reported with steroids and local anesthetic infiltration of anomalous lumbosacral articulations in eight out of 10 patients, but only one patient remained pain-free after two years [9]. For patients with BS who fail to respond to conservative management, surgical excision of the anomalous joint is indicated. Jönsson, et al. reported symptomatic relief in nine out of 11 patients who had an injection of local anesthetic into the anomalous articulation; however, eventually they had to undergo resection of the accessory joint for permanent relief [10].

## Conclusions

This syndrome must be considered as a differential diagnosis for LBP in young people. A thorough evaluation is required to identify the cause of LBP in young patients, which includes a detailed history, physical examination, and radiological imaging to confirm the diagnosis. Failure to follow this protocol will lead to misdiagnosis and suboptimal management.
